# Aerobic Fitness as an Important Moderator Risk Factor for Loneliness in Physically Trained Older People: An Explanatory Case Study Using Machine Learning

**DOI:** 10.3390/life13061374

**Published:** 2023-06-12

**Authors:** Samuel Encarnação, Paula Vaz, Álvaro Fortunato, Pedro Forte, Cátia Vaz, António Miguel Monteiro

**Affiliations:** 1 Department of Sport Sciences, Instituto Politécnico de Bragança (IPB), 5300-253 Bragança, Portugal; samuel01.encarnacao@gmail.com (S.E.);; 2 Research Centre in Basic Education (CIEB), Instituto Politécnico de Bragança (IPB), 5300-253 Bragança, Portugal; 3 Department of Pysical Activity and Sport Sciences, Universidad Autónoma de Madrid (UAM), Ciudad Universitaria de Cantoblanco, 28049 Madrid, Spain; 4 Research Centre in Sports Sciences, Health Sciences and Human Development (CIDESD), 5001-801 Vila Real, Portugal; 5 CI-ISCE, Higher Institute of Educational Sciences of the Douro (ISCE Douro), 4560-708 Penafiel, Portugal; 6 Department of Education and Supervision, Instituto Politécnico de Bragança (IPB), 5300-253 Bragança, Portugal

**Keywords:** quality of life, well-being, cardiorespiratory fitness, mental health, aging, artificial intelligence

## Abstract

Background: Loneliness in older people seems to have emerged as an increasingly prevalent social problem. Objective: To apply a machine learning (ML) algorithm to the task of understanding the influence of sociodemographic variables, physical fitness, physical activity levels (PAL), and sedentary behavior (SB) on the loneliness feelings of physically trained older people. Materials and Methods: The UCLA loneliness scale was used to evaluate loneliness, the Functional Fitness Test Battery was used to evaluate the correlation of sociodemographic variables, physical fitness, PAL, and SB in the loneliness feelings scores of 23 trained older people (19 women and 4 men). For this purpose, a naive Bayes ML algorithm was applied. Results: After analysis, we inferred that aerobic fitness (AF), hand grip strength (HG), and upper limb strength (ULS) comprised the most relevant variables panel to cause high participant loneliness with 100% accuracy and F-1 score. Conclusions: The naive Bayes algorithm with leave-one-out cross-validation (LOOCV) predicted loneliness in trained older with a high precision. In addition, AF was the most potent variable in reducing loneliness risk.

## 1. Introduction

In the middle of the 21st century, global society seems to continue to equate aging as a concern that we will have to address in the future. However, that future has now arrived and, in fact, as cited by Rodrigues et al. [1] (p. 1) “population aging is no longer a matter of the future, but of the present”, with population aging very close to becoming one of the most significant social transformations of the 21st century. This is according to the United Nations (UN) Regional Information Center for Western Europe (2023), which predicts the doubling of the number of older people aged 60 years or more by 2050 and points to Europe as currently having the highest percentage of its population in this age group (25%). There seems to be evidence that one of the changes related to aging is the emergence of loneliness. Several risk factors are listed in the literature for loneliness: moments of transition throughout life, children leaving their parents’ home, loss of a spouse, retirement, divorce, autonomy, social support, and health deterioration [2,3,4]. 

According to Pocinho et al. [5], considering a cognitive approach to loneliness, it is necessary to take into account, on the one hand, the type of social relationships that an in-dividual has and, on the other hand, the level of social contacts that he/she wishes to have. Therefore, loneliness emerges in the discrepancy between actual and desired social contacts [5,6]. However, it is important to note that loneliness is not the same as objective and voluntary isolation, i.e., a person who voluntarily isolates themself to perform activities such as meditating or working to produce more at certain times of their life, is not necessarily, by this option, a person who feels loneliness. Similarly, while it is true that loneliness is not synonymous with objective isolation, such as living alone or having few social interactions, one must realize that people who interact with others can be alone and experience loneliness [6].

In terms of population aging within society and within the dynamics and organization of the family, loneliness seems to have emerged as a social problem that is increasingly prevalent and that deserves our particular attention, given the consequences that may arise from it. These occur at various levels, can lead to suffering for those who experience it and are associated with a lower quality of life [1], increased mortality, a higher risk of some cardiovascular, metabolic, and neurological diseases [1,6], and a higher risk of metabolic syndrome, functional disability, dementia, and even mild cognitive impairment. It also affects mental and emotional health. At this level, it should be noted that loneliness has been associated with depression, decreased well-being, anxiety, and suicidal ideation, but it is also essential to point out that it can put older people in a situation of greater susceptibility to abuse by others [6].

Although feelings of loneliness can occur at all ages, they tend to be more prevalent in people aged 60 and older. A study conducted by Yang and Victor [7] in 14 European countries observed that, in Portugal, loneliness increases with age, with a prevalence of 14.9% in people aged over 60 years. This is higher than in Nordic countries where the increase is not so pronounced with aging, perhaps due to both the existence of active aging policies and the greater integration and participation of older people in society. Of the 14 countries studied, in all but one the prevalence of loneliness is higher in people aged 60 and over compared with the two lower age groups (under 30 and between 30 and 59) [7].

The World Health Organization (WHO)’s Global Strategy and Action Plan on Ageing and Health [8] seeks to develop age-friendly environments that help promote healthy aging and the maintenance of functional capacity through the interaction of physical and mental abilities, allowing for the wellbeing of the older person. Thus, the WHO suggests a set of coordinated actions to maximize older people’s participation, promote their autonomy and involvement, and improve different domains of functional capacities, such as building and maintaining relationships [8]. 

These actions emerge as fundamental when reflecting on the issue of loneliness and its impact on older people. The sustainable development goals of the UN 2030 Agenda—specifically goal number 3, quality health—refer to the need to ensure access to quality health, to promote the wellbeing of people of all ages and to promote mental health and wellbeing [9]. Pels and Kleinert [10], in a literature review of 37 studies, concluded that physical activity level (PAL) has significant potential in reducing loneliness, although this successful decrease in loneliness was also influenced by social engagement. In addition, Pels and Kleinert’s review considered habitual physical activity despite the presence of regular physical exercise interventions in most of the included studies [10]. In this way, studies that investigate physical exercise’s protective effects on older people’s mental-health-related outcomes are relevant to the applied practice. 

Furthermore, clarifying the concepts of physical activities (PA), and physical exercise is important because they share similarities and differences in their constructs, per the WHO [11]. Second, and as per the WHO, PA can be comprehended as “any bodily movement produced by skeletal muscles that requires energy expenditure”. In addition, the WHO states that PA that is more intense than habitual PA is required to increase energy expenditure. Reducing SB has been determined to protect against obesity, metabolic syndrome, cardiovascular disease, type II diabetes, and other associated non-communicable chronic diseases [12]. Additionally, in 1985 Caspersen et al. [13] described physical exercise as “a subset of physical activity that is planned, structured, and repetitive and has as a final or an intermediate objective the improvement or improvement or maintenance of physical fitness”.

After this positioning, other organizations have clarified and recalled the differences between the two concepts [14,15,16]. In fact, there is strong evidence from metanalysis showing that PAL contributes significantly to both physical and mental health, as it is able to improve cardiovascular fitness [17] and life satisfaction [18], and reduce incidences of anxiety and depression in older people [19]. Conversely, physical exercise, due to its tendency to be incremental in terms of training variables i.e., volume, intensity, and session density, can significantly increase the amount of high-intensity PA i.e., elevated energy expenditure, which in turn acts to stimulate the positive effects on the body multisystem, inclusive of mental health [1,20,21,22].

According to Hawkley [6], reducing loneliness involves implementing joined-up approaches at the individual, community, and societal levels. Thus, considering a physical exercise program’s potential as a protective factor when considering loneliness in older people is legitimate. It is important, then, to reflect on a program’s impact and contribution to the establishment of healthy social relationships among its participants. Additionally, improving functional fitness and elevating high-intensity PAL beyond habitual PAL is important for physical and mental health [23].

In this context, artificial intelligence (AI) has emerged as a relevant advance in actual scientific research, and is being used efficiently in several knowledge areas [24,25,26]. AI can understand and precisely interpret applied situations of real life by mimicking human brain learning patterns [27]. In health sciences, AI is extremely useful for screening health conditions in a timely manner and has the capacity to generalize results. 

For instance, in the mental health field, AI helps professionals to build more targeted strategies to prevent and reduce mental health damage in older people [28,29]. Thus, with its precise inferences, it should be used as an important tool to help health policies apply public resources more correctly when developing time-efficient preventive campaigns in both local and national instances [29,30]. However, regarding the state-of-the-art knowledge recorded in our literature review, at this present moment we did not find any research utilizing an ML analysis that integrates older people’s physical fitness and loneliness. For this purpose, we aimed to apply an ML algorithm to understand the influence of sociodemographic variables, physical fitness, PAL, and SB on the loneliness feelings of physically trained older people. Based on previous evidence about the positive effect of physical fitness on the mental health of the elderly [31,32,33,34,35,36,37,38,39,40], we hypothesize that physical fitness will be more relevant than sociodemographic variables in predicting trained older people’s loneliness feelings.

## 2. Materials and Methods

This is an explanatory case study, with measurements made in a cross-sectional de-lineation, which aims to understand the influence of sociodemographic variables, physical fitness, PAL, and SB on the loneliness feelings of physically trained older people. For this main purpose, we relied upon evaluations of several morphofunctional measures (muscle strength/power, AF, upper limb flexibility, body composition, basal metabolism, SB, and PAL), and we selected the older peoples’ loneliness feelings scores as the dependent target variable.

### 2.1. Dataset

For convenience, we selected 23 older people (19 women and 4 men, aged between 66–76 years old, with body weight and height 158 ± 6 cm), from the project called “+Idade + Saúde” present in the High School of Education of the Polytechnic Institute of Bragança (IPB). The data collection occurred on January 2023, at the IPB. The participants had performed a multicomponent training intervention (MCT) one year before the data collection. The MCT was characterized by a five-minute warmup aiming to prepare the full body for the main training part. The participants performed twelve resistance exercises directed to the major muscle groups, followed by 10 min of dynamic and static balance exercises, finagling with 10 min of high-intensity interval training (HIIT), and completed 40 min for the total training session. The training sessions occurred on Mondays, Tuesdays, and Fridays, from 9 to 10 pm at the gym from the IPB’s High School of Education. Trained physical exercise coaches supervised all MCT sessions. The coaches controlled the training intensity of all training sessions using the Borg Category Ratio 10 (CR-10) scale of perceived exertion, ranging from 1–10, adopted from Borg [41]. We limited the training intensity to a maximum of 7–8 points on the scale (moderate to intense training intensity), aiming to reduce the excessive and more dangerous physical efforts during the intervention period. 

**Figure 1:**
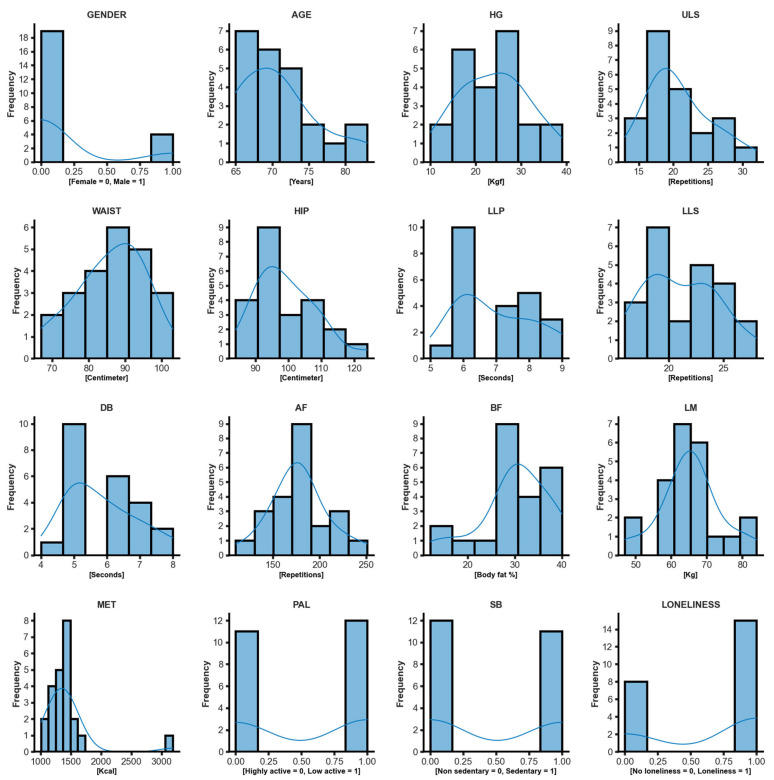
Data visualization of the participant’s gender, age, physical fitness, body composition, and loneliness scores. Frequency, absolute participants frequency; MET, basal metabolism.

We described the participant’s characteristics in mean and standard deviation (M ± SD) for the variables gender, age (years), anthropometric variables (height in centimeters (cm), body weight in kilogram (kg), lean muscle mass (kg), basal metabolism in kilocalories (kcal), HG kilogram-force (kgf), and AF (step repetitions)) and these are indicated in Table 1. In addition, we describe the frequency of participants on the variables gender, age, AF, HG, upper limb strength (ULS), waist circumference, hip circumference, lower limb muscle power (LLP), lower limb strength (LLS), DB, body fat percentage (BF), lean mass (LM), metabolism (MET), PAL, SB and loneliness in the histogram shown in Figure 1. With the chi-square test of simple proportions (X^2^), we verified the homogeneity of the participant’s sociodemographic categoric variables (sex (male or female), scholarly level (< or >9 years of school), marital status (married, single, divorced or widow), if the participant lives alone, economic status (≥ or <2 monthly salaries), religion status (religious or not), smoker, alcohol consumer, SB, PAL, loneliness feelings scores, body mass index (BMI) obesity classifications, waist circumference, hypertension, diabetes, cholesterol, cardiovascular disease, stroke, column pain, respiratory disease, arthrosis, osteoporosis, and labyrinthitis), indicated Table 2 [26,42,43].

### 2.2. Ethical Aspects

This study was approved by the ethics committee of research with humans from the Higher Institute of Educational Sciences of the Douro, with a register number (No: 2.576). The study was conducted in accordance with the Declaration of Helsinki.

### 2.3. Sample Measurements

In this study we measured variables related to body composition, sociodemographics, the older person’s physical fitness, basal metabolism, PAL, SB, and loneliness feelings scores. All protocols are detailed and described below.

#### 2.3.1. Body Composition

Body composition was measured using a phase-sensitive, double-frequency bioelectrical impedance analysis (BIA) scale (Tanita, DC430-PMA), with a precision of 0.1 kg. Then, the BIA provided absolute and percentual lean body mass (stratified by body sections (upper and lower limb)), fat mass, body water content, visceral fat (absolute score), and total metabolism (kcal). Before the measurement, each subject removed their clothing and metal jewelry and rested supine for 5 min to equilibrate body fluids. The participant’s lower limbs were positioned open approximately 45° to the median body. The participant’s upper limbs were positioned 30° from the trunk holding the double-frequency device. Hip and waist circumference were measured in cm with a metallic anthropometric tape (Cerscorf, copyright) with a precision of 0.01 m.

#### 2.3.2. Sociodemographic Variables

The sociodemographic questionnaire was applied to measure the variables (sex, scholarly level, marital status, if the participant lives alone, economic status, religion status, smoker, alcohol consumption, loneliness feelings scores, BMI obesity classifications, waist circumference, hypertension, diabetes, cholesterol, cardiovascular disease, stroke, column pain, respiratory disease, arthrosis, osteoporosis, and labyrinthitis).

#### 2.3.3. Hand Grip Strength

HG was measured through a digital palmar dynamometer, CAMRY^®^, Portugal, adopting as a metric the maximal kgf reached with a palm grip. The older person stays standing, with arms not touching the trunk, and at the signal of the researcher, the older person must exert the maximal palm grip on the dynamometer for four seconds [44]. Two attempts were given, and the evaluator recorded the best result for each participant.

#### 2.3.4. Upper Limb Strength 

ULS was measured with the arm curl test [42]. The participants were positioned in a chair 43 cm high, holding a 2 kg dumbbell (woman’s standard weight). At the evaluator’s signal, the participants performed as many elbow flexions and extensions as they could for 30 s, and the maximal number of repetitions was recorded.

#### 2.3.5. Lower Limb Power 

LLP was measured through the five-time sit-to-stand test [45]. The test was performed in a standardized chair of 0.49 m in height. The evaluator set their stopwatch when the participants lost contact with the chair, and the participants performed five repetitions as quickly as possible, the evaluator stopping the stopwatch at the end of the fifth repetition when the participants sat for the fifth time in their chair. The evaluator stimulated the participants throughout the test to ensure that they were always performing at the maximum speed of movement and technique. Two attempts were performed with an interval of 60 s between each attempt, and the shortest time was noted [45].

#### 2.3.6. Lower Limb Muscle Strength 

LLS was measured with the seat-to-stand test, from the “Functional Fitness Test Battery” [42]. The participants were positioned standing up in front of a 43 cm high chair. At the evaluator’s signal, the participants performed the movement of sitting and standing up as many times as possible for 30 s. The number of repetitions was recorded.

#### 2.3.7. Dynamic Balance

DB was evaluated with the time-up-and-go test, from the “Functional Fitness Test Battery” [42]. The participants were positioned in a chair 43 cm high, facing a cone at 2.44 m. At the evaluator’s signal, the participants walked as fast as possible, going around the cone, and returning to the initial position. The participants performed two attempts, and the evaluator recorded the shortest time.

#### 2.3.8. Aerobic Fitness

AF was evaluated through the two-minute step test, from the “Functional Fitness Test Battery” [42]. The evaluator requested each older individual to perform the maximal amounts of steps in the same place for two minutes. Indeed, the researcher advised the older persons to stop only if “it is really necessary”, aiming to extract the maximal performance of each older person during the test. After the voice signal “Ready…go!!”, the older persons started performing maximal steps during the test time. The evaluator recorded the number of repetitions performed for both legs.

#### 2.3.9. Upper Limb Flexibility

ULF was measured with the back scratch test, from the “Functional Fitness Test Battery” [42]. The evaluator instructed the participants to place one hand over the shoulder, reach the other hand, place it underneath, and find the fingers or overlap one hand with the other behind the back. The evaluator recorded the number of cm between the tips of the middle fingers. A positive distance was attributed when the participants overlapped one hand over the other, zero distance (equal to zero) when they touched one hand to the other, and negative distance when the participants did not reach their hands.

### 2.4. Physical Activity Levels and Sedentary Behavior

We measured PAL and SB using a Fitbit^®^ smartwatch, model Inspire 2, validated by Silva et al. [46] to measure PAL and SB. This was placed in the participant’s fist and was used uninterrupted for 7 days (a normal week). The researcher advised the older subjects not to remove the smartwatch at any moment of the day, aiming to record the weekly PAL as precisely as possible. As a measurement metric, the total daily number of steps was considered in order to verify whether the participants accomplished the minimal 7000 steps per day. In so doing, we used the references from the study of Locke et al. [47] and thus classifying those participants with less than 7000 steps per day as “negative” (0) and those with more than 7000 steps per day as “positive” (1) [12]. SB was recorded when the participants remained still for more than 15 min in a lying or reclining position, with the watch beginning to record as of 15 min and onwards. We considered the threshold of 6–8 h/day total siting time as the SB cutoff for health risks, following the recommendations identified in the meta-analysis of Patterson et al. [43].

#### Loneliness Feelings Scores

The loneliness feelings scores were verified through the UCLA loneliness scale, developed and revised by Russel et al. [48,49,50] and validated for older Portuguese people [5]. This consists of 20 items that can evaluate an older person’s loneliness through a four-point Likert scale (1 = never, 2 = rarely, 3 = sometimes, 4 = frequently), which together measure the frequency of loneliness feelings. The range of punctuation on the scale is 14 to 78 points, and when a subject presents a score ≥32 points on the scale, it is considered an elevated risk of loneliness. Thus, we classified the participants that scored ≤32 as “negative” for loneliness (0), and those that presented scores ≥32 as “positive” (1) [5].

## 3. Naive-Byes Artificial Intelligence Algorithm Application 

**Figure 2:**
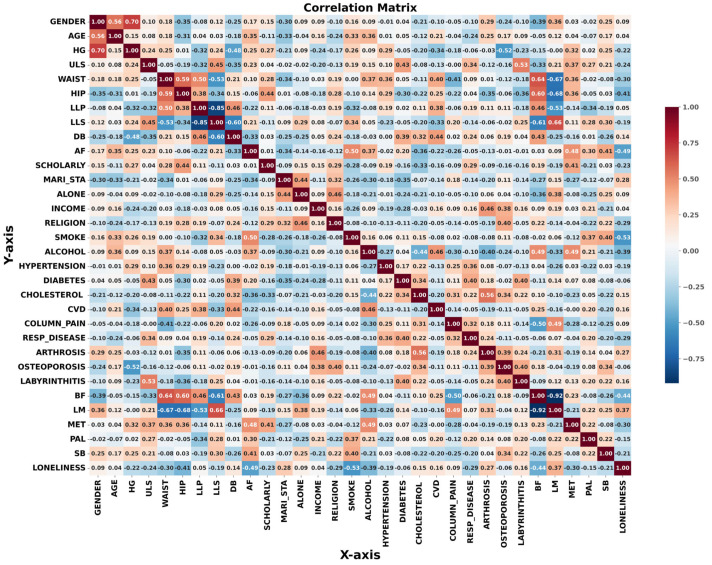
Correlation matrix of the study’s variables.

Procedures were performed using the Python^TM^, version 3.11.3, programing language [51], where the libraries “numpy”, “matplotlib.pyplot”, and “pandas” were imported to work with the csv archive. This made it possible to manipulate and organize the dataset. Indeed, the “sklearn” library was also imported, activating all of the algorithm’s requirements and the model fit’s processing functions. Initially, we performed an empirical search with the sociodemographic variables (gender (male = 1, female = 0), age (years), HG (kg), ULS through arm-curl test (repetitions), lower limb power through the five-time seat-to-stand test (repetitions), lower limb muscle strength through the sit-to-stand test 30 s (repetitions), dynamic balance through the time-up-and-go test (seconds), AF through the two-minute step test (walk repetitions), level of education (more than 9 years = 0, lower than 9 years = 1), marital status (married = 0, widower = 1), live alone (accompanied = 0, alone = 1), income (≥two wage per month = 0, ≤than one wage per month = 1), religious (yes = 0 no = 1), smoker (yes = 1, no = 0), alcohol (yes = 1, no = 0), hypertension (yes = 1, no = 0), diabetes (yes = 1, no = 0), deregulated cholesterol (yes = 1, no = 0), cardiovascular disease (CVD) (yes = 1, no = 0), stroke (yes = 1, no = 0), column pain (yes = 1, no = 0), respiratory disease (1 = yes, no = 0), arthrosis (yes = 1, no = 0), osteoporosis (yes = 1, no = 0), labyrinthitis (yes = 1, no = 0), body fat mass (percentual values), muscle mass (kilograms), basal metabolism total kilocalories, PAL (highly active (yes = 0, no = 1), and SB (highly sedentary = 1, not sedentary = 0)) which we arranged as explanatory variables in an array “*x*”. In addition, we placed the target variable “loneliness” in an array “*y*”. The correlation matrix of all variables is shown in Figure 2, adopting effect size cutoffs of ≤0.10—very small, ≥0.20—moderate, ≥0.30—large, and ≥0.40—very large, according to the assumptions of Funder et al. [33]. 

Beyond this, because the dataset size (*n* = 23) is considered very small and with further target variable characteristics (dichotomous numeric variable), we adopted naive-Byes as the aimed analysis algorithm. In addition, we performed the LOOCV during the naive-Bayes analysis. LOOCV is widely used to evaluate the algorithm performance, helping to measure the analysis generalization level (applied practice value). LOOCV allow researchers to use all datasets for both algorithm testing and training actions, thus making the most of the available information [52]. Naive-Bayes is based on Bayes’ theorem, which can learn and interpret the probability of an event’s occurrence, assuming that a variable presented in a conjunct of data has any relationship with any other variable [26,53]. For this purpose, naive-Bayes is understood by:

(1) $$P\left(Y|X\right)=\frac{P\left(X|Y\right)P\left(Y\right)}{P\left(X\right)}$$

where, for propositioning of this theorem, *Y* and *X* are considered as:

1. *P* (*Y*), the “prior probability”, is the initial degree of belief in *Y*.
2. *P* (*Y*|*X*), the “posterior probability”, is the degree of belief having accounted for *X*. It is interpreted as “the probability of *Y*, given that *X* is the case”.
3. The quotient *P*(*X*|*Y*)/*P*(*X*) represents the support *X* provides for *Y*.

Furthermore, due to its simplicity and precision in working with small datasets, this model is considered very applicable to the health sciences, leading to precise and reliable diagnosis and classification of several conditions where other more complex ML or deep learning analyses cannot understand the data [54,55,56]. 

## 4. Results

We performed the naive-Bayes with LOOCV for the first time using the function “train_test_index”, placing 100% of the dataset for training and 100% for testing to investigate the influence of all of the explanatory variables on the older person’s loneliness feelings scores. Considering all variables for the model, we have a low accuracy (40%) and a low F-1 score (42%). 

Next, we carefully take out the low coefficient correlations (Spearman’s r < 0.20) or meaningless correlations (age, gender, income, scholarly, live alone, marital status, religion, smoke, alcohol, column pain, osteoporosis, arthrosis, labyrinthitis, CVD, respiratory disease, cholesterol, diabetes, hypertension, body fat percentage, lean mass, basal metabolism, waist circumference, hip circumference, LLS, DB, SB and PAL). After each variable is removed, we retest the algorithm, and achieve a performance of only 65% accuracy and 43% F-1 score. However, when we run the algorithm with the remaining variables (HG, ULS, and AF) in the x array and loneliness feelings in the y array, we achieve 100% accuracy and F-1 score.

**Figure 3:**
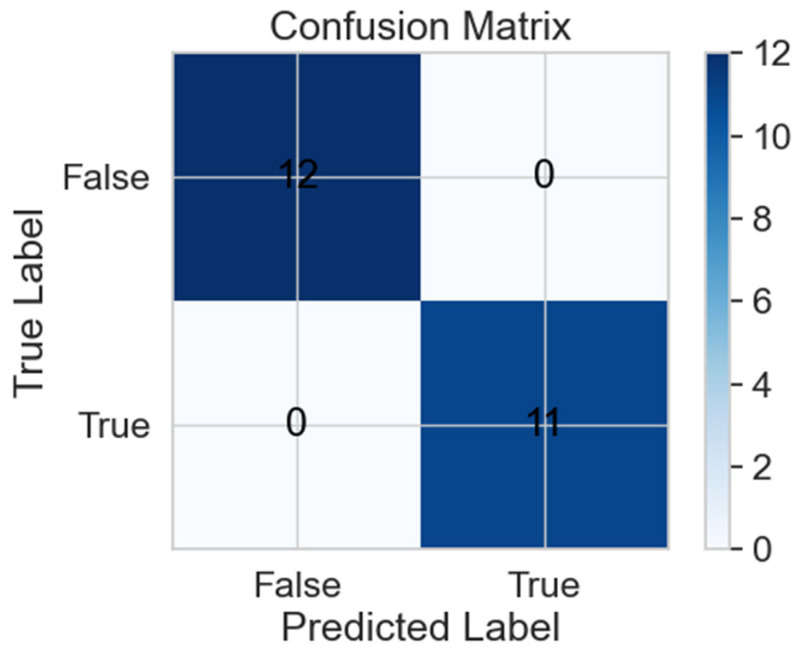
Confusion matrix for test prediction. 48% of the total dataset (*n* = 11) in predicted label and 52% (*n* = 12) are described in absolute values. “False” in the *x* and *y* axis corresponds to the false native and false positive outcomes. Similarly, “True” in the *x* and *y* axis corresponds to true positive and true native outcomes found per AI analysis.

We describe the naive-Bayes algorithm’s predicted and true outputs in Figure 3. 

## 5. Discussion

This study aimed to apply an ML algorithm to understand the influence of sociodemographic variables, physical fitness, PAL, and SB on physically trained older people’s loneliness feelings. Our hypotheses were confirmed when the naive-Bayes algorithm with LOOCV showed high clinical validity (100% accuracy and F-1 score) and understanding of the true positives and negatives in the model. Further, we obtained the most efficient variables panel when combining PAL, ULS, and HG as the loneliness predictors, where AF presented the more significant coefficient of correlation followed by ULS and HG. These results are very interesting because they show that the participants’ physical training status was more important than sociodemographic factors or PAL in reducing their loneliness risk. Loneliness in old age is highly associated with early physical disability and early mortality. In fact, identifying the loneliness-influencing factors in old age and in different circumstances is a global health policy challenge that must be overcome in order to promote efficient preventive interventions [57].

In a survey study with 6652 older people, Musich et al. [58] verified that moderate-to-high physical activities were responsible for 15–30% less loneliness and social isolation likelihoods in their participants. Physical exercise, especially aerobic exercise ≥60% of maximum oxygen consumption (VO_2_ max) increments the endorphins release [31]. Endorphins are neurochemical molecules that improve mood and wellbeing by producing essential neurotransmitters, such as serotonin and norepinephrine, that indirectly influence perceived feelings of loneliness [31]. Moreover, and similarly to our findings, in a randomized controlled trial, Mouquinho et al. [32] found that both moderate and high-intensity physical exercise provoked improvements in stress, anxiety, depression, and resilience of healthy adults during the 2019 coronavirus pandemic confinement. Interestingly, Mouquinho et al. [32] also found that HIIT was more effective in promoting these changes. Furthermore, Lampinen et al. [33], in a longitudinal cohort study, accompanied 663 older people (441 women) from 1988 to 1996 (eight-year follow-up), and noted that age-related exercise intensity decreases were associated with higher depression risk in the participants. Our results, supported by the current literature, show that higher-intensity physical activity (preferably regular physical exercise) is more effective than lower-intensity physical activity to positively affects an older person’s mental health with regard to loneliness feelings.

In addition, in a one-year follow-up cohort study, undertaken by Lee and Park [34] identified that moderate to vigorous physical activity was protective against depressive symptoms in older people. Furthermore, Lee and Park identified that the higher physical activity intensity benefited the most depressive participants. Furthermore, in a meta-analysis of experimental studies, Korman et al. [35] perceived that both MCT and HIIT improved the depression index in older people with several mental illness degrees. In addition, they noted that HIIT was more potent in reducing the participant’s depression scores. In the present study, the MCT had a high aerobic and oxidative profile, characterized by an HIIT block after balance and resistance training blocks during all training sessions. This should help to understand why AF contributes more to the prediction of loneliness feelings in the participants. This information is strengthened in the meta-analysis of Wu et al. [59], which found that HIIT was more efficient than moderate-intensity continuous training in improving older people’s cardiorespiratory fitness. 

Another mental-health-related physiological mechanism modulated by physical exercise is the cortisol released by the hypothalamic–hypnotizes–adrenal axis. In a complex manner, physical exercise acutely increases blood cortisol concentrations, indicating high physiologic stress; however, persistent exercise leads to a reaction from the immune system, generating stress-resistance adaptations and stimulating hermetic hypothalamic–hypnotizes–adrenal axis regulation [36,37]. Due to the regulated physiological stress via cortisol release reduction, a homeostasis of neurotransmitter release occurs, controlling mood, sleep, and reducing anxiety and depression risk [60]. Moreover, corroborating our results, Lucertini et al. [38] found a significant and inverse association between the daily cortisol release and cardiorespiratory fitness in 22 older men. Therefore, higher cortisol levels are associated with loneliness feelings in older people [61].

Therefore, HG was significantly and inversely correlated with an older person’s loneliness feelings. HG strength is a physical function capacity that is positively related to overall health in different life phases, correlating with early all-cause mortality and thus considered a valuable physiological functioning metric [62]. Moreover, HG is also associated with mental health; specifically in older people. It is related to improved self-efficacy and confidence when performing important activities of daily living (ADLs), such as carrying shopping or undertaking housework. Thus, physical autonomy and an independence mindset are positively related to cognition and well-being [63]. Agreeing with our results, in a twelve-year longitudinal and prospective study with 2570 older people, Kim and Park [63] verified that loneliness significantly correlated with the participant’s HG decreases over time; indeed, these associations were more profound in men than women. 

Moreover, in another prospective study, with a total dataset of 6118 participants from 2004/2005, 2008/2009, and 2012/2013, Vingeliene et al. [64] verified that HG positively correlated with an older person’s loneliness score. However, these results are significant for men and women younger than 80 years old [64]. Furthermore, in another prospective study, Torres et al. [65] compared data from the Brazilian Longitudinal Study of Aging (ELSI-Brazil), (2019–2021; *n* = 6929) with the Longitudinal Study of Aging (2018–2019; *n* = 5902) and identified a negative association between HG in Brazilian women. Strengthening our results, Tavares et al. [66] analyzed a dataset from 137 older people (70 females) living in a Portugal primary health care house, and found an inverse correlation between HG and the participant’s fragility index, showing the higher mental and physical health risks due to lower HG values.

Moreover, ULS presented a significant and inverse correlation with the older person’s feelings of loneliness. ULS at an older age is also related to improved performance in ADLs, such as carrying objects independently, participating in social activities such as dancing and playing instruments, or performing leisure tasks that require coordination and manual control [67]. Regarding mental health, in a cross-sectional observational study with 200 older Japanese participants (124 women), Tajika et al. [40] noted that upper limb overall strength and capability were inversely correlated with the participant’s depressive symptoms. In addition, in a cross-sectional study with 1201 adults ≥50 years old from Ghana, Gyasi et al. [68] identified an increased fragility risk in the participants with reduced ULS. Furthermore, in a cross-sectional study with 259 older people (155 females), Furtado et al. [69] identified that a high physical function status, which included upper limb capability, was significantly related to the older person’s mental health and quality of life perception index. Therefore there are direct associations between physical fitness and an older person’s mental health and loneliness feelings; a high physical independence allows greater social and physical exercise engagement, which is relevant for mental health, wellbeing, and loneliness feelings [10]. Similarly to our results, Schrempft et al. [70] analyzed 267 older people of both sex and perceived significantly reduced moderate and high PAL in the groups with increased loneliness feelings. In addition, McAuley et al. [71] found that loneliness changes are related to the changes in the participant’s social support perception. McAuley et al. [71] found that participants who felt greater support from other course members became less lonely. These findings seem to concur with the idea that older people must develop self-determined motivation, i.e., to feel autonomous, competent, and interrelated to the surrounding social context [1]. Our study strengthened this evidence when the sociodemographic variables of living alone, marital and economic status and scholarly level were discarded as valid predictive factors. Despite these results, the study participants performed MCT in a group class for one year before the analysis, strengthening social contacts and friendly feelings and providing possibilities to improve cognition and mental health [72]. Despite these positive aspects, our study’s participants presented high loneliness feelings scores (*n* = 16, 70%), which emphasizes the urgency of promoting interventional strategies for older people’s mental health.

### 5.1. Strengths and Limitations

Some limitations must be declared in regard to the above research. First, the limited data set size (*n* = 23) cannot totally represent the influencing factors, such as marital status, living alone, scholarly level and income. This led to lower coefficient correlations in the data bank and worsened the algorithm performance. Second, our dataset had women in as its majority (*n* = 19, 82%). Third, we analyzed only trained and socially engaged older people, which does not fully reflect the overall population. Fourth, there was a lack of mental health-related biochemical marker analyses, which would help to better understand the contribution of physical fitness contribution to the older person’s loneliness feelings. Fifth, loneliness feelings are a continuous mental health condition, and we performed only cross-sectional analyses, limiting the observations in longitudinal changes. Furthermore, these present results may change when applying the algorithm to other contexts. In this sense, we suggest that these results must be interpreted carefully. We highlight the successful naive-bayes with 100% predictive precision as the primary study’s strength. Based on our preliminary results, we suggest that researchers from different sociocultural contexts of Portugal and other high- or low-income countries could perform similar ML analyses in different older populations (trained, physically inactive, healthy, or bearers of some pathological condition) looking to interpret the influence of physical exercise practice in different environmental conditions. In addition, researchers should consider performing ML analyses with larger cohorts and in longitudinal designs to increase the strength of the evidence.

### 5.2. Practical Applications

Through this case study, we provide important knowledge for different health professionals, such as physical exercise coaches, psychologists, psychiatrists, and even basic health system professionals, who may consider the benefits of exercise for older peoples’ mental health and loneliness feelings when guiding their patients. Physical exercise coaches in particular could take advantage of this knowledge to develop physical exercise interventions based on responsive variables. Finally, these professionals must consider systematized and progressive physical exercise training as a powerful tool to prevent mental health risks in older people. We also suggest that group classes are essential for the maintenance of older peoples’ wellbeing.

## 6. Conclusions

The naive-Bayes AI algorithm had high precision when predicting loneliness feelings in trained older people. In addition, we found AF, HG, and ULS together formed the best predictive variables panel. Finally, AF was found to be the most potent variable to be inversely related to older peoples’ loneliness feelings.

## Figures and Tables

**Table 1:** Participant’s characteristics.

Variables	M	SD
Age (years)	71	5
Height (cm)	158	6
Body weight (kg)	65	10
Lean muscle mass (kg)	5	9
Basal metabolism (kcal)	1427	473
HG (kgf)	24	8
AF (step repetitions)	178	31

Note—M, mean; SD, standard deviation; cm, centimeter; kg, kilogram; kcal, kilocalories; kgf, kilogram-force.

**Table 2:** Sociodemographic characteristics of the sample.

Demographics	N	%	*p*-Value
Female sex	19	82%	0.001
Scholarly level (<9 Years of School)	8	34%	0.01
Marital status (single or divorced or widow)	7	30%	0.001
Live alone	4	17%	0.001
Income (<2 monthly Wages)	15	65%	NS
Religion status (religious)	22	96%	0.001
Smoker	3	13%	0.001
Drink alcohol	4	17%	0.001
Sedentary behavior	11	48%	NS
Low physical activity levels	12	52%	NS
**Medical conditions**			
Loneliness	16	70%	0.04
BMI overweight (>25 Kg/M^2^ for men—26.6 Kg/M^2^ for women)	12	52%	NS
Waist circumference (≥97 cm for men—≥88 cm for women)	11	48%	NS
Hypertension	6	26%	0.001
Diabetes	5	22%	0.001
Deregulated cholesterol	11	48%	NS
Cardiovascular disease	1	4%	0.001
Stroke	0	0%	0.001
Column pain	7	30%	0.001
Respiratory disease	1	4%	0.001
Arthrosis	10	43%	NS
Osteoporosis	5	22%	0.001
Labyrinthitis	1	4%	0.001

Note—N, total observations for each variable. %, percentage values; *p*-value; level of significance of 95%. NS, non-significant.

## Data Availability

The data presented in this study can be shared up on request for the authors.
